# Three Inulin-Type Fructans from *Codonopsis pilosula* (Franch.) Nannf. Roots and Their Prebiotic Activity on *Bifidobacterium longum*

**DOI:** 10.3390/molecules23123123

**Published:** 2018-11-29

**Authors:** Jiankuan Li, Xin Zhang, Lingya Cao, Jiaojiao Ji, Jianping Gao

**Affiliations:** 1 School of Pharmaceutical Science, Shanxi Medical University, Taiyuan 030001, China; jiankuanli@sxmu.edu.cn (J.L.); zhangxin201689@163.com (X.Z.); caolingyablue2008@163.com (L.C.); jijiao963@163.com (J.J.); 2 The Engineering Technology Research Center of Authentic Herbal Material Resources Development of Shanxi Province, Shanxi Medical University, Taiyuan 030001, China; 3 School of Basic Medical Science, Shanxi Medical University, Taiyuan 030001, China

**Keywords:** *Codonopsis pilosula* (Franch.) Nannf., inulin, prebiotic activity, polysaccharide, *Bifidobacterium longum*

## Abstract

Radix Codonopsis, derived from the roots of *Codonopsis pilosula* (Franch.) Nannf., *Codonopsis pilosula* (Franch.) Nannf. Var. *modesta* (Nannf.) L.T. Shen and *Codonopsis tangshen* Oliv., has been used as traditional Chinese medicine for improving poor gastrointestinal function, treating gastric ulcers and chronic gastritis in China. Inulin-type fructans are carbohydrates consisting mainly of β (2→1) fructosyl-fructose links in chemical structure and exhibit a range of properties such as prebiotic activity, fat substitutes in low-calorie foods and disease-modifying effects. The prebiotic effects of inulin-type fructans are hypothesized to improve gastrointestinal function through alterations to gut microbiota composition and metabolism. In the present study, three inulin-type fructans with high degree of polymerization (DP = 16, 22, and 31) were isolated from the roots of *Codonopsis pilosula* (Franch.) Nannf. and their structures were confirmed by MALDI-TOF-MS, 1D- and 2D-NMR. The prebiotic activity of these fructans was evaluated by detecting growth stimulation on *Bifidobacterium longum*. The results demonstrated that three fructans at a concentration of 2.0 g/L exhibited significant growth stimulation on *Bifidobacterium longum* in a time-dependent manner (*p* < 0.01). The data indicated that inulin-type fructans in Radix Codonopsis could be used as potential prebiotics.

## 1. Introduction

Radix Codonopsis, derived from the roots of *Codonopsis pilosula* (Franch.) Nannf., *Codonopsis pilosula* (Franch.) Nannf. Var. *modesta* (Nannf.) L.T. Shen and *Codonopsis tangshen* Oliv., has been used in traditional Chinese medicine for improving poor gastrointestinal function, treating gastric ulcers and chronic gastritis in China [1,2]. Phytochemical studies indicate that polysaccharides, phenylpropanoids, polyacetylenes and alkaloids are the main components in Radix Codonopsis [1,2]. Recently, several polysaccharides have been obtained from Radix Codonopsis, some of which exhibit potential bioactivities [3,4,5,6,7,8].

Fructans are fructose polymers that are consisted of one or more fructosyl-fructose link in chemical structure. Inulin-type fructans are fructans that have mostly or exclusively β (2→1) fructosyl-fructose linkages and usually terminate with only one glucose unit through an α-d-glucopyranosyl bond, and that exhibit in linear molecules. Because of fructose in inulin-type fructans existing in β configuration, inulin-type fructans are prevented from hydrolysis by human digestive enzymes in the gastrointestinal tract and usually fermented to produce short-chain carboxylic acids such as acetate, butyrate and propionate during their passage through the gastrointestinal tract [9,10]. Some inulin-type fructans have been evaluated to exhibit diverse functions such as regulation of blood sugar and lipid, gastrointestinal health, anticancer and regulation of human gut microbiota [11,12]. Recently inulin-type fructans have been reported to have a wide range of pharmaceutical applications such as stabilization of proteins, modified drug delivery and disease-modifying effects [13,14].

Previously, we reported an inulin-type fructan from *Codonopsis pilosula* (Franch.) Nannf. roots and its anti-fastric ulcer effects [15]. Recently, Fu and his colleagues reported a mixture of inulin-type fructans (DP = 2–17) from *Codonopsis pilosula* Nannf. Var. *modesta* (Nannf.) L.T. Shen and its prebiotic activity on *Lactobacillus* [16]. The present research reports three inulin-type fructans isolated from the dried roots of *Codonopsis pilosula* (Franch.) Nannf. and their prebiotic activity by detecting growth stimulation on *Bifidobacterium longum*.

## 2. Results and Discussion

### 2.1. Structure Identification of Fructan ***1***, ***2*** and ***3***

**Figure 1:**
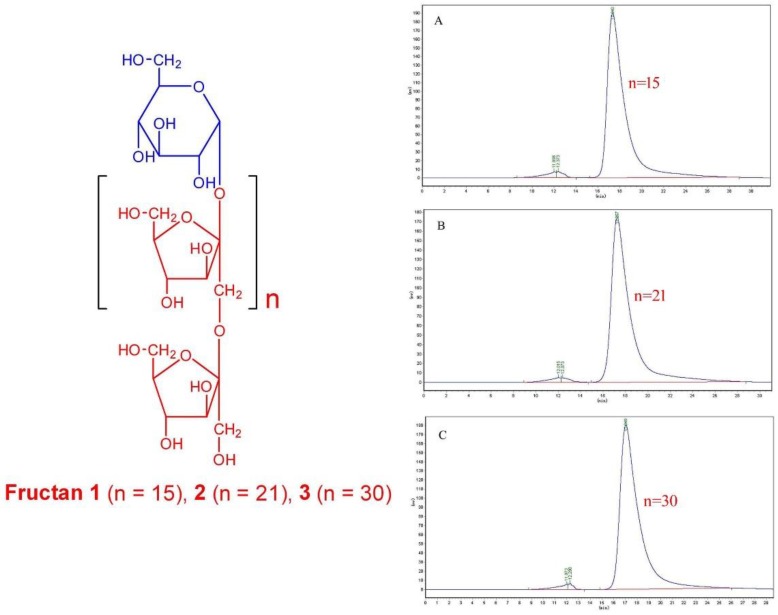
The structure and HPGPC curves of fructan **1**(**A**), **2**(**B**) and **3**(**C**).

As shown in Figure 1, the High performance gel permeation chromatography (HPGPC) of fructan **1** (A), **2** (B) and **3** (C) exhibited a single and symmetrically sharp peaks at 17.341 min (**1**), 17.257 min (**2**) and 17.040 min (**3**), respectively, which indicated that fructan **1**, **2** and **3** were homogeneous oligosaccharides According to the equation of standard molecular weight (lg*M*_p_ = −0.5322*t*_R_ + 12.618), the molecular weights (*M*p) of fructan 1, 2 and 3 were estimated to be 2400, 2700 and 3500, respectively.

**Figure 2:**
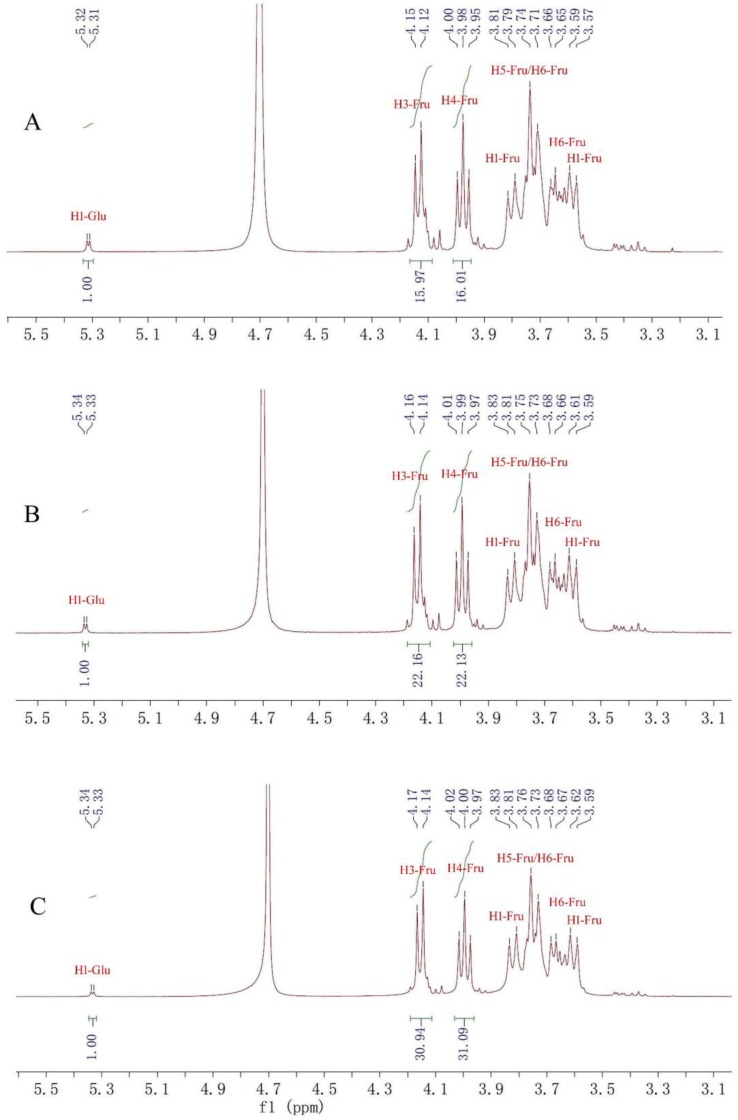
^1^H-NMR spectra of fructans **1**(**A**), **2**(**B**) and **3**(**C**).

**Figure 3:**
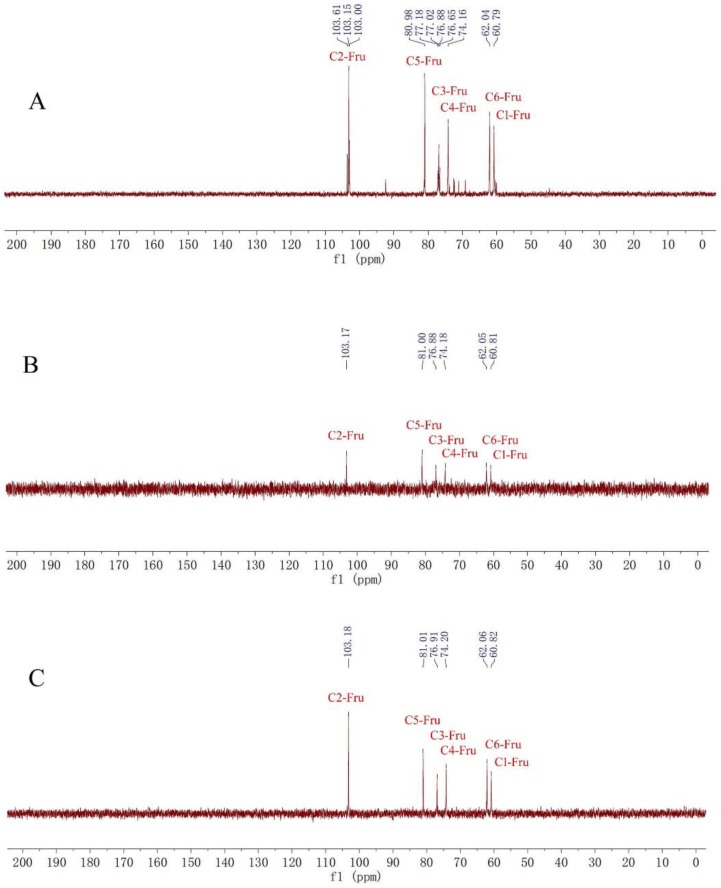
^13^C spectra of fructans **1**(**A**),**2**(**B**) and **3**(**C**).

**Figure 4:**
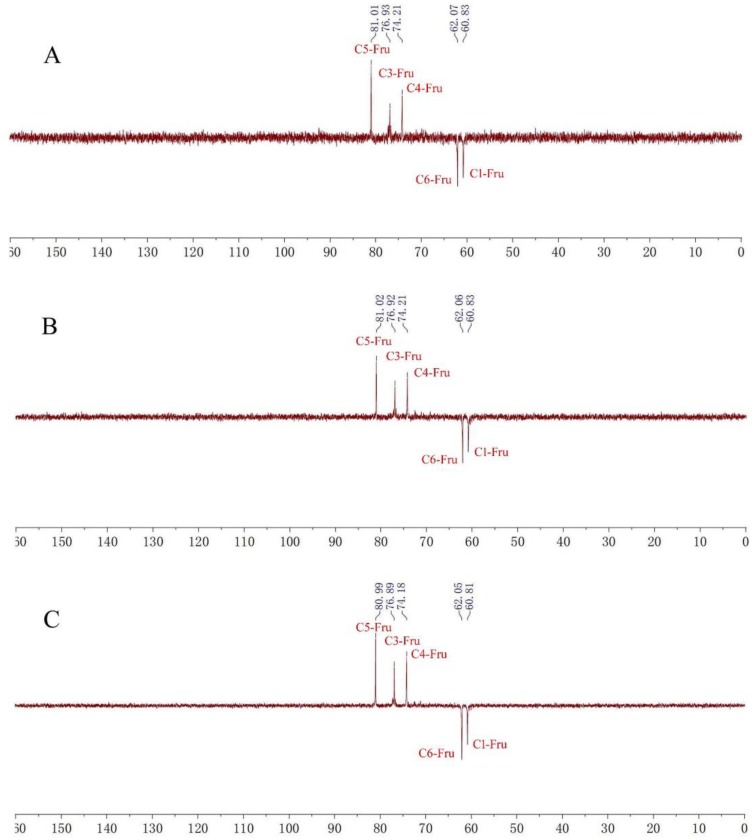
DEPT135 spectra of fructans **1**(**A**), **2**(**B**) and **3**(**C**).

The ^1^H-NMR spectra of fructan **1** (A), **2** (B) and **3** (C) (Figure 2) showed similar hydrogen signals occurring from 6.00 ppm to 3.00 ppm. The only difference was that the integration ratio of H signal at about 5.3 ppm (Glu-1H) to H signal at about 4.1 ppm (Fru-H3) or 4.0 ppm (Fru-H4), which was used to calculate the degree of polymerization (DP) of inulin-type fructans. Therefore, the DPs of fructans **1**, **2** and **3** were confirmed to be 16, 22 and 31, respectively. In the ^13^C-NMR spectra of fructans **1**, **2** and **3** (Figure 3), the three fructans also exhibited similar signals occurring from 105 ppm to 60 ppm, which were confirmed as one quaternary carbon at about 103 ppm, three tertiary carbons at about 81 ppm, 76 ppm and 74 ppm and two secondary carbon at about 62 ppm and 60 ppm according to DEPT 135 spectra (Figure 4), which were consistent with the HSQC spectrum (Supplement Material) and were used for the complete assignment of H and C signals (Table 1). In HMBC spectra (Supplement Material), the correlation between C2-Fru/H1-Fru and C2-Fru/H1-Glu could be observed, which indicated the presence of (2→1)-linked β-d-fructofuranosyl and terminal glucose. ^1^H- and ^13^C-NMR chemical shifts were identified with data from the published literature and similar to the inulin-type fructan standard spectrum [17,18,19,20]. Therefore, the structures of fructan **1**, **2** and were completely identified, as shown in Figure 1.

**Figure 5:**
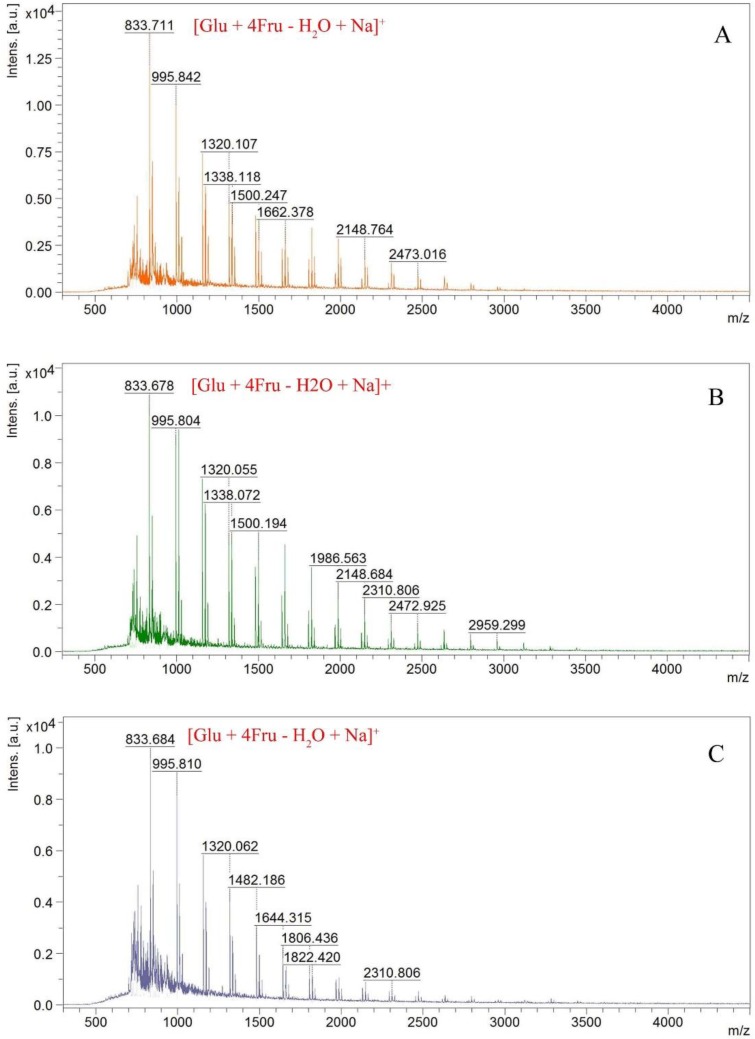
MALDI-TOF mass spectra of fructan **1**(**A**), **2**(**B**) and **3**(**C**).

The MALDI-TOF mass spectra of fructans **1**, **2** and **3** (Figure 5) were similar to that of an inulin-type fructan and there was a mass difference of 162 between two neighboring ions, which corresponded to fructan/fructan residues [17,21,22]. The ion with maximum abundance at *m*/*z* 833 was assumed to be [Glu + 4Fru − H_2_O + Na]^+^.

All data described above are in accordance with the characteristic data of inulin-type fructans, so fructans **1**, **2** and **3** were confirmed to be α-d-glucopyranosyl-(1→2)-(β-d-fructofuranosyl)_n_-(1→2)-β-d-fructofuranoside (n = 15, 21, 30, respectively).

### 2.2. Prebiotic Activity of Fructan ***1***, ***2*** and ***3***

**Figure 6:**
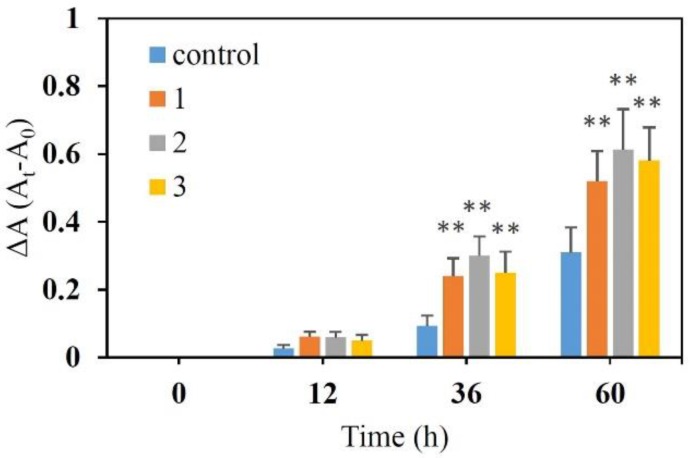
Growth stimulation of fructan **1**, **2** and **3** on *Bifidobacterium longum*. ** *p* < 0.01 compared to the control.

Bifidobacteria, naturally present in the dominant colonic microbiota, represent up to 25% of the cultivable fecal bacteria in adults [23]. The health benefits of bifidobacterial are reflected in the commonly accepted definition of prebiotics: food ingredients that exhibit the capacity of selectively stimulating the growth of gut bacteria including bifidobacterial and lactobacilli [24,25]. Bifidobacteria can utilize a variety of carbohydrates as energy substrates for growth, including monosaccharides, oligosaccharides, and polysaccharides [26]. Recently, the prebiotic effects of inulin-type fructans are hypothesized to improve gastrointestinal function through alterations to gut microbiota composition and metabolism. Valcheva and his colleagues reported that inulin-type fructans (n = 12 and 13) increased gut Bifidobacteriaceae and Lachnospiraceae abundance and improved gut function in ulcerative colitis patients [27]. The study by Azpiroz and his coworkers indicated that Chicory-derived inulin-type fructans promoted bifidobacterial growth and improved gut function in patients with abdominal symptoms and reduced tolerance of intestinal gas [28]. Meanwhile, Wilson and Whelan reviewed in detail the functions and applications of inulin-type fructans in gastrointestinal disorders [29]. In the present study, the prebiotic effect of fructans was evaluated by detection of growth simulation on *Bifidobacterium longum*. The result (Figure 6) showed that fructans **1**, **2** and **3** at concentration of 2.0 g/L exhibited significant growth stimulation on *Bifidobacterium longum* compared with the control in a time-dependent manner (*p* < 0.01), which indicated that fructan **1**, **2** and **3** could be used by *Bifidobacterium longum* to improve growth. Meanwhile, there were no significant differences between fructans **1**, **2** and **3**.

## 3. Material and Methods

### 3.1. Materials and Chemicals

D101 macroporous resin was obtained fromChengdu Kelong Chemical Reagent Factory(Chengdu, China). MCI GEL CHP 20P HIGH POROUS POLYMER was purchased from Mitsubishi Chemical Corporation(Tokyo, Japan). Other chemical regents were purchased from Damao Chemical Regent Factory (Tianjin, China). Nuclear magnetic resonance (NMR) analysis was conducted on an AVANCE III 400 MHz (Bruker, Karlsruhe, Germany). Mass spectra (MS) were performed on an ultraflex MALDI-TOF MS (Bruker, Karlsruhe, Germany). Dextran 2700, 5250, 9750, 13050, 36800, 64650, 135350 were obtained from National Institute for the Control of Pharmaceutical and Biological Products (Beijin, China). *Bifidobacterium longum* ATCC15707 was purchased from Guangdong Microbial Species Conservation Center (Guangzhou, China).

The roots of *Codonopsis pilosula* (Franch.) Nannf. were collected when the plant had grown for two years, from Pingshun county in Shanxi province, China in July 2015, when the local temperature was 23 ± 5 °C. The roots were identified by Professor Jianping Gao. The specimen (201512-01-MCM) was kept at School of Pharmaceutical Science, Shanxi Medical University.

### 3.2. Extraction and Isolation

The dried roots of *Codonopsis pilosula* (20 kg) were crushed and extracted two times with methanol (10:1, *v*/*w*) by ultrasonic extraction at room temperature for 1 h. The extract was concentrated in vacuum and was suspended in water, which was followed by extraction successively by ethyl acetate and *n*-butanol to obtain the ethyl acetate fraction (130 g), *n*-butanol fraction (180 g) and water fraction (350 g). The water fraction was subjected to D101 macroporous resin column chromatography eluted with water to obtain Fraction I (36 g) and Fraction II (54 g) according to phenol-sulfuric acid method by determination of ultraviolet absorption of eluents [30]. Fraction II was subjected to MCI (CHP 20P) rein column chromatography eluted with H_2_O and detected by phenol-sulfuric acid method to obtain fructan **1** (60 mg). Fraction I was subjected to MCI CHP 20P column chromatography eluted with water to obtain fructan **2** (85 mg) and **3** (62 mg) according to the phenol-sulfuric acid method. The sevage method was used to remove proteins from frutan **1**, **2** and **3**.

### 3.3. Homogeneity and Molecular Weight Determination

The homogeneity and molecular weight of fructans **1**, **2** and **3** were determined by high performance gel permeation chromatography (HPGPC) performed with a LC-10AT HPLC system (SHIMADZU, Kytot, Japan) fitted with a Tskgel G4000 PWXL column (300 mm × 7.8 mm, Particle Size: 10 μm) and a Shodex RI-20H refractive index detector. The mobile phase was ultrapure water and the flow rate was 0.3 mL/min at 35 °C. The molecular weight of fructans **1**, **2** and **3** were estimated with reference to a calibration curve obtained from a set of Dextran standards of known molecular weight (Dextran 70, 180, 2500, 4600, 7100, 10,000, Purity: above 99%).

### 3.4. NMR and TOF-MS Analysis of Fructan **1**, **2** and **3**

Dried fructans **1**, **2** and **3** (10 mg, respectively) were dissolved in D_2_O in 5-mm NMR tube and measured for ^1^H-NMR, ^13^C-NMR, DEPT, HSQC and HMBC on an AV-400 spectrometer (Bruker, Rheinstetten, Germany). The TOF-MS was conducted using an Ultraflex MALDI-TOF/TOF spectrometer (Bruker Daltonics, Billerica, MA, USA). The spectra were obtained in positive-mode.

Fructan **1**: white solid, ^1^H-NMR (400 MHz, D_2_O) δ: 5.31 (1H, d, *J* = 4 Hz), 4.12 (16H, d, *J* = 12 Hz), 3.98 (16H, t, *J* = 12 Hz), 3.57–3.81 (80H, m). ^13^C-NMR (100 MHz, D_2_O) δ: 103.15, 80.98, 76.88, 74.16, 62.04, 60.79.

Fructan **2**: white solid, ^1^H-NMR (400 MHz, D2O) δ: 5.33 (1H, d, *J* = 4 Hz), 4.14 (22H, d, *J* = 12 Hz), 3.97 (22H, t, *J* = 12 Hz), 3.59–3.83 (110H, m). ^13^C-NMR (100 MHz, D_2_O) δ: 103.17, 81.00, 76.88, 74.18, 62.05, 60.81.

Fructan **3**: white solid, ^1^H-NMR (400 MHz, D_2_O) δ: 5.33 (1H, d, *J* = 4 Hz), 4.14 (31H, d, *J* = 12 Hz), 4.00 (31H, t, *J* = 12 Hz), 3.59–3.83 (155H, m). ^13^C-NMR (100 MHz, D_2_O) δ: 103.18, 81.01, 76.91, 74.20, 62.06, 60.82.

### 3.5. Prebiotic Test

*Bifidobacterium longum* was grown on BBL agar basal medium (Qingdao Hope Biotechnology Co. LTD, Qingdao, China) in an anaerobic chamber at 37 °C for 48 h. A single colony was then removed in TPY liquid medium (Qingdao Hope Biotechnology Co. LTD, Qingdao, China) mainly containing (g/L) peptone 5.0, yeast extract 10.0, NaCl 2.0, K_2_HPO_4_ 1.0, KH_2_PO_4_ 1.0, MgSO_4_ 7H_2_O 0.4, CaCl_2_ 0.2, NaHCO_3_ 10.0, Tween 80 1.0 mL, L-cysteine 0.5, pH 7.2. TPY liquid medium was supplemented with 2.0 g/L fructans **1**, **2** and **3** respectively, as a sole carbon source. The TPY medium without any carbon sources represented the negative control. *Bifidobacterium longum* suspensions were incubated at 37 °C for 12 h, 36 h, and 60 h in an anaerobic chamber. Culture growth was evaluated by measuring the optical density of the tested medium (Varioskan Flash 525 Microplate Reader, Thermo Fisher Scientific, Waltham, MA, USA). The growth was evaluated as the change in absorbance (ΔA = A_t (initial)_−A_0 (final)_) at a wavelength of 600 nm after 12 h, 36 h, and 60 h. Experiments were carried out in triplicate.

### 3.6. Statistical Analysis

Each experiment was performed at least three times. All data are expressed as the mean ± S.D. Statistical analysis was performed by Student’s *t*-test using Microsoft Office Excel 2013 software. Difference at *p* < 0.01 was indicated to be statistically significant.

## 4. Conclusions

Three inulin-type fructans (**1**, **2** and **3**) were isolated from the roots of *Codonopsis pilosula* (Franch.) Nannf. and their structures were confirmed as α-d-glucopyranosyl-(1→2)-(β-d-fructofuranosyl)_n_-(1→2)-β-d-fructofuranoside (n = 15, 21, 30, respectively) by MALDI-TOF-MS, 1D- and 2D-NMR. The prebiotic activities of fructans **1**, **2** and **3** were evaluated by detecting growth stimulation on *Bifidobacterium longum*. The results demonstrated that fructans **1**, **2** and **3** at concentration of 2.0 g/L exhibited significant growth stimulation on *Bifidobacterium longum* in a time-dependent manner (*p* < 0.01). These data indicated that the inulin-type fructans in Radix Codonopsis could be used as potential prebiotics.

## Figures and Tables

**Table 1:** ^1^H- (400 MHz, D_2_O) and ^13^C-NMR (100 MHz, D_2_O) chemical shifts in ppm for fructan **1**, **2** and **3**.

	Fructan 1		Fructan 2		Fructan 3	
No. of C/H	^1^H	^13^C	^1^H	^13^C	^1^H	^13^C
1	3.79, 3.59	60.79	3.81, 3.61	60.81	3.81, 3.62	6.82
2	-	103.15	-	103.17	-	103.18
3	4.12	76.88	4.16	76.88	4.17	76.91
4	3.98	74.16	3.99	74.18	4.00	74.20
5	3.74	80.98	3.75	81.00	3.76	81.01
6	3.71, 3.66	62.04	3.73, 3.66	62.05	3.73, 3.67	62.06

## Supplementary Materials

The following are available online. S1–S6: HSQC and HMBC spectra of Fructan **1**–**3**.

## Abbreviations

The following abbreviations are used in this manuscript:

DEPT	distortionless enhancement by polarization transfer
DP	degree of polymerization
HMBC	heteronuclear multiple bond correlation
HPGPC	high performance gel permeation chromatography
HSQC	heteronuclear singular quantum correlation
MS	mass spectrometer
NMR	nuclear magnetic resonance spectrometer
