# Supplementary material for: Three Inulin-Type Fructans from Codonopsis pilosula (Franch.) Nannf. Roots and Their Prebiotic Activity on Bifidobacterium longum
Source: Molecules. 2018 Nov 29;23(12):3123. doi: 10.3390/molecules23123123 (PMC6320984; doi:10.3390/molecules23123123)
Supplement: Supplementary file 1 [file molecules-23-03123-s001.pdf]

Inulin-Type Fructans from *Codonopsis pilosula* (Franch.) Nannf. Roots and Their Prebiotic Activity on *Bifidobacterium longum*

Jiankuan Li[a, b], Xin Zhang[a], Lingya Cao[a, c], Jiaojiao Ji[a, b], Jianping Gao*[a, b]

[a] School of Pharmaceutical Science, Shanxi Medical University, Taiyuan 030001, China; jiankuanli@sxmu.edu.cn (J. Li); zhangxin201689@163.com (X. Zhang); caolingyablue2008@163.com (L. Cao); jijiao963@163.com (J. Ji)

[b] The Engineering Technology Research Center of Authentic Herbal Material Resources Development of Shanxi Province, Shanxi Medical University, Taiyuan 030001, China

[c] School of Basic Medical Science, Shanxi Medical University, Taiyuan 030001, China

*Corrspondence: jpgao123@163.com; Tel: +86-351-3985-244

Content

S1: HSQC specrtum of Fructan 1.

S2: HMBC specrtum of Fructan 1.

S3: HSQC specrtum of Fructan 2.

S4: HMBC specrtum of Fructan 2.

S5: HSQC specrtum of Fructan 3.

S6: HMBC specrtum of Fructan 3.

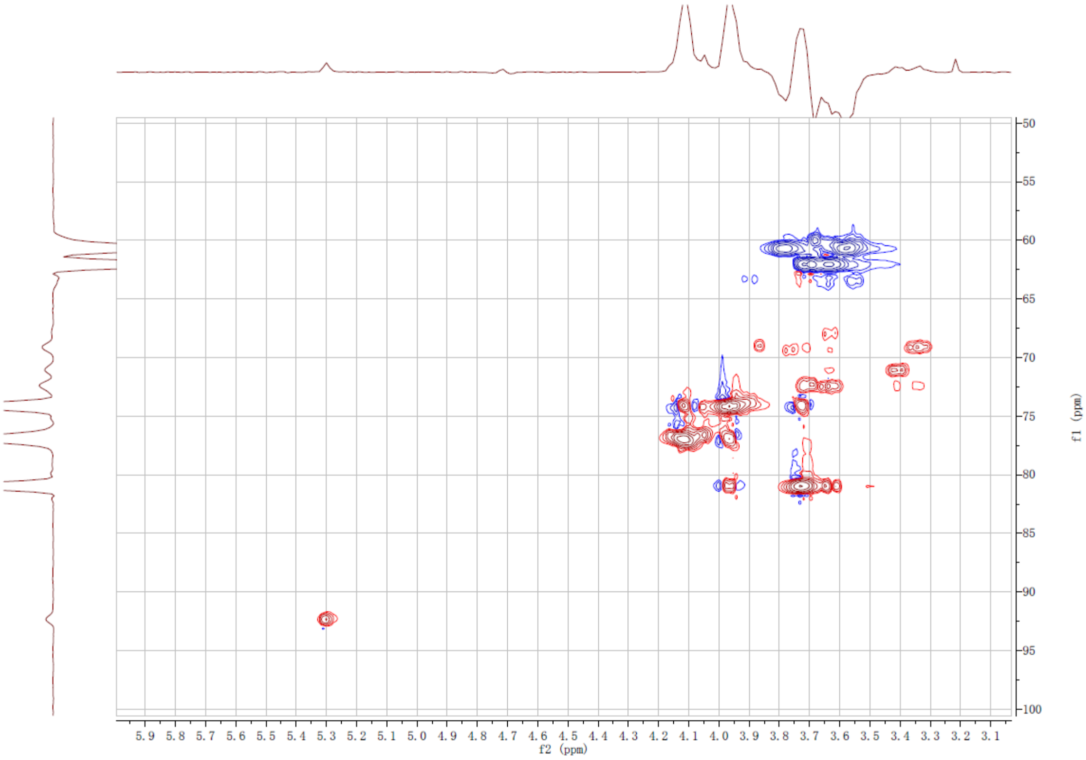

S1: HSQC specrtum of Fructan 1.

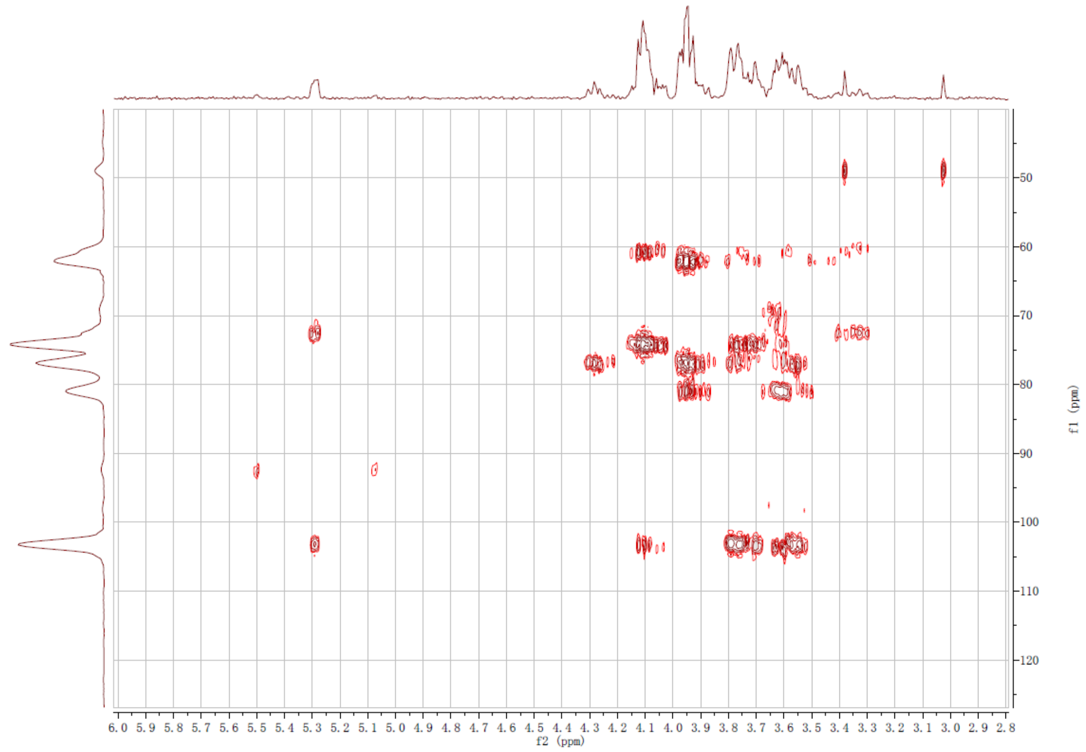

S2: HMBC specrtum of Fructan 1.

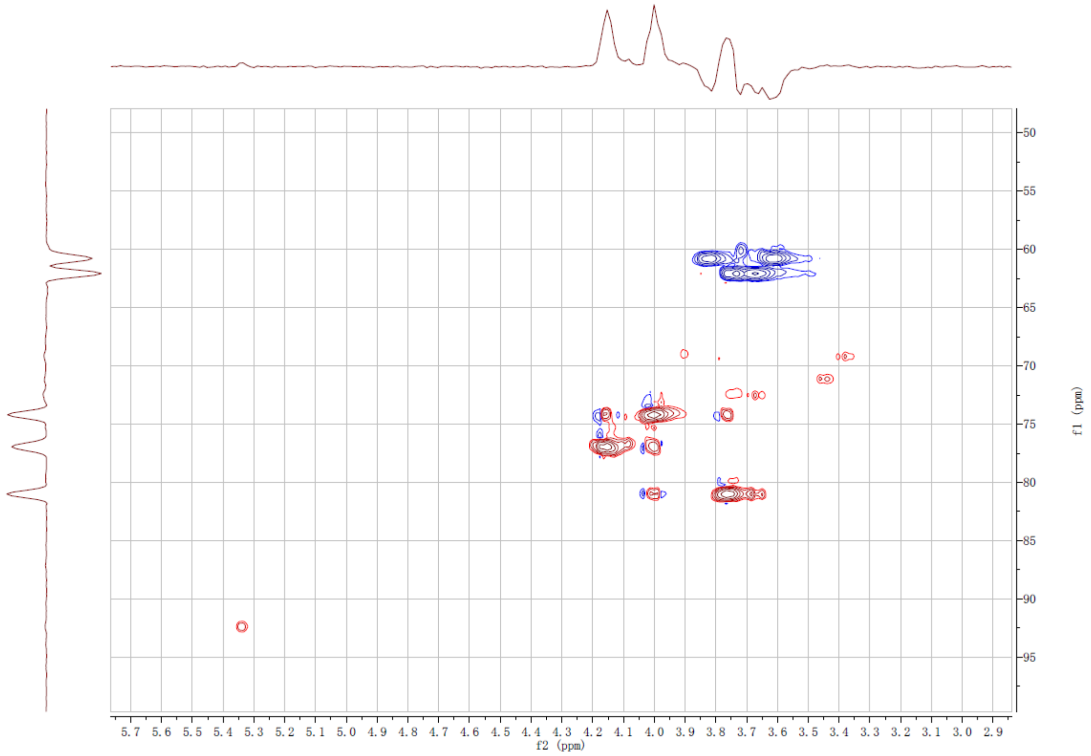

S3: HSQC specrtum of Fructan 2.

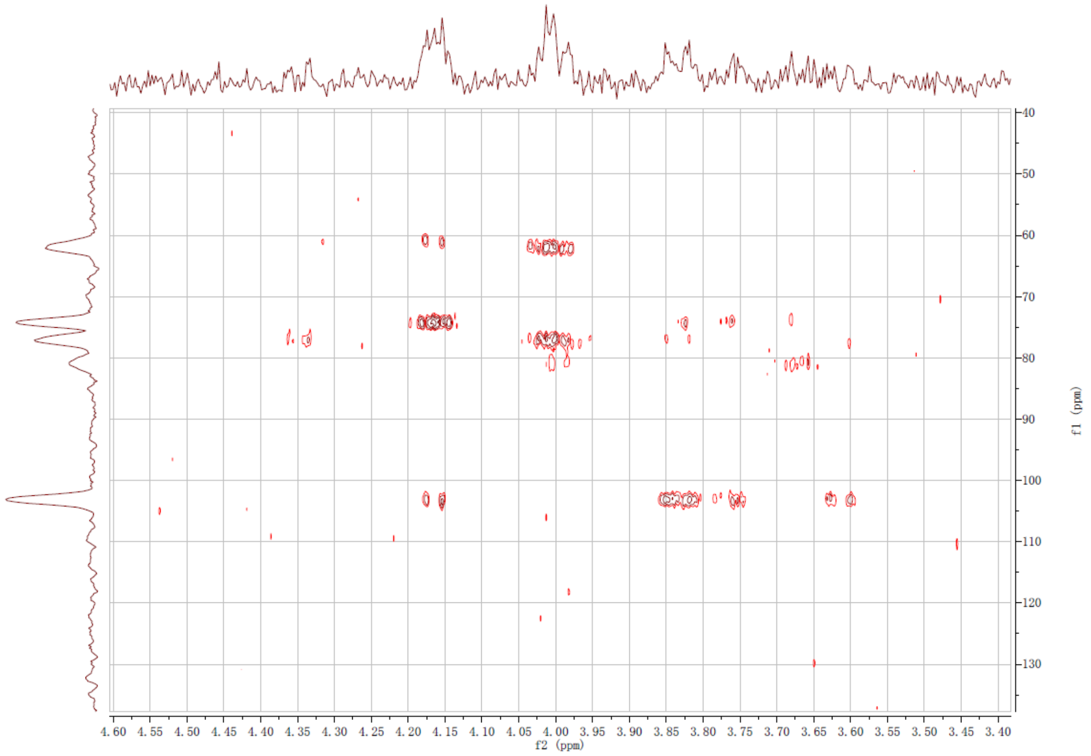

S4: HMBC specrtum of Fructan 2.

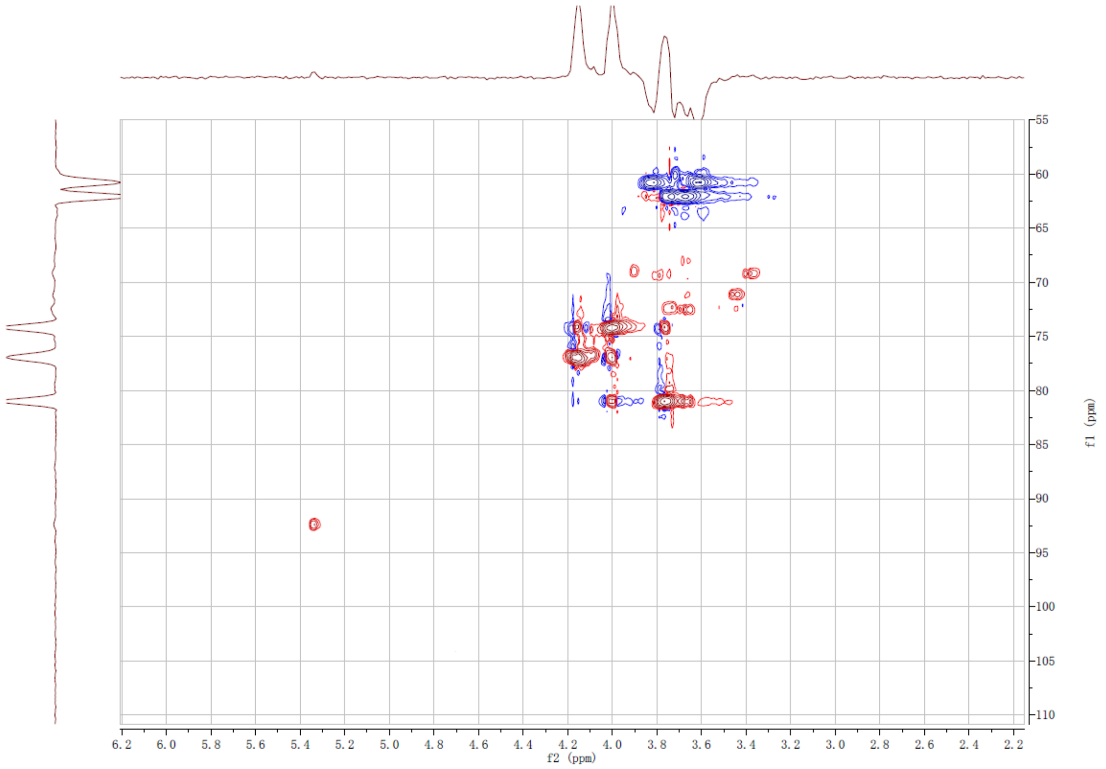

S5: HSQC specrtum of Fructan 3.

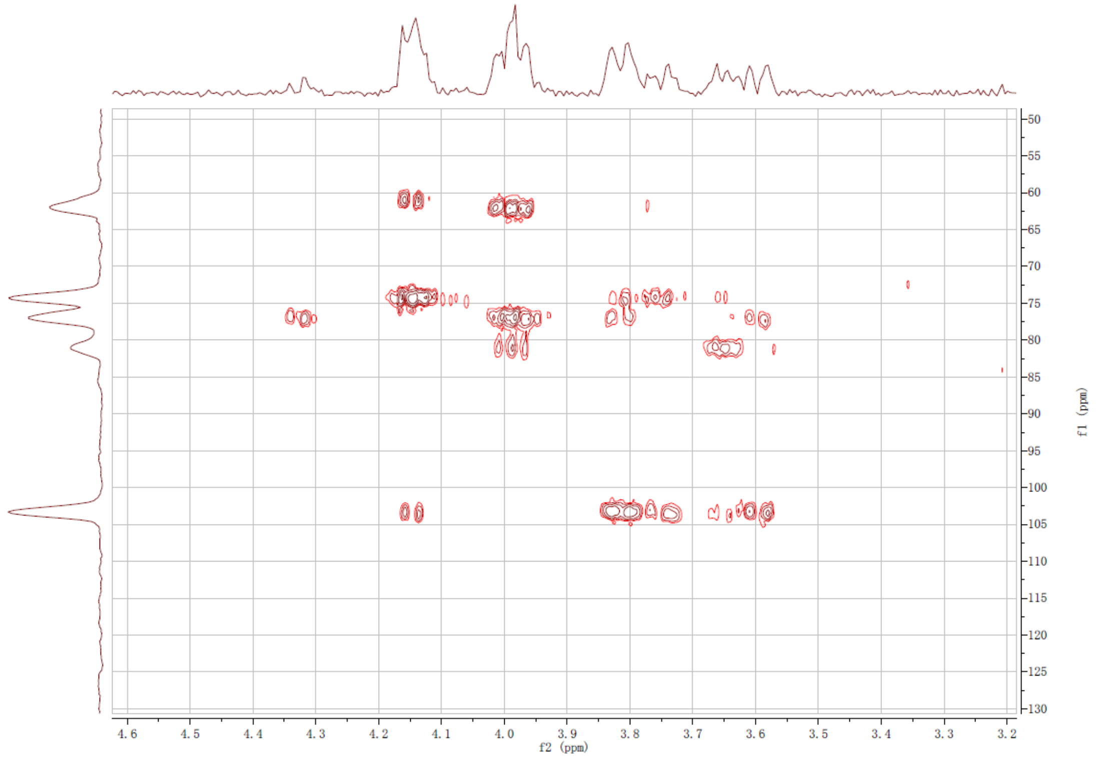

S6: HMBC specrtum of Fructan 3.
